# Commentary on “Food addiction, orthorexia nervosa and dietary diversity among Bangladeshi university students: a large online survey during the COVID-19 pandemic”

**DOI:** 10.1186/s40337-023-00812-0

**Published:** 2023-05-20

**Authors:** André Eduardo da Silva Júnior, Mateus de Lima Macena, Dafiny Rodrigues Silva Praxedes, Nassib Bezerra Bueno

**Affiliations:** 1 Escola Paulista de Medicina, Universidade Federal de São Paulo, São Paulo, SP 04023-062 Brazil; 2 Laboratório de Nutrição e Metabolismo (LANUM), Faculdade de Nutrição, Universidade Federal de Alagoas, Maceió, AL Brazil

**Keywords:** YFAS, Food addiction, COVID-19, SARS-CoV-2

## Abstract

The food addiction construct is receiving increasing attention from researchers and clinicians worldwide. Given its rise, scientific production on the subject is increasingly abundant. Conducting studies evaluating food addiction in emerging countries is of great importance, given that most scientific production comes from high-income countries. A recent study aimed to explore the prevalences of orthorexia nervosa and food addiction and their associations with dietary diversity in university students in Bangladesh during the COVID-19 pandemic. This correspondence presents questions about using the older version of the modified Yale Food Addiction Scale to assess food addiction. It also highlights issues related to the prevalence of food addiction observed in the study.

## Main text

We have read the article entitled “Food addiction, orthorexia nervosa and dietary diversity among Bangladeshi university students: a large online survey during the COVID-19 pandemic” published by Sultana et al. [1] in the prestigious journal. Sultana et al. conducted an exciting study that explored the prevalences of orthorexia nervosa and food addiction (FA) and their associations with dietary diversity in university students in Bangladesh during the COVID-19 pandemic. We recognize that developing research in emerging countries is extremely important for producing knowledge about the FA construct, given that most scientific production on this topic comes from high-income countries [2]. Despite this, the study by Sultana et al. has some points that deserve attention and that need to be considered in interpreting its results.

The Yale Food Addiction Scale (YFAS) and its versions are the main and most used tools to determine FA worldwide [3]. The first version of the YFAS and its shortened version, the modified Yale Food Addiction Scale (mYFAS), were developed taking into account the diagnostic criteria for substance dependence from the Diagnostic and Statistical Manual of Mental Disorders (DSM) - IV. However, in 2013, the DSM-5 was released and proposed significant changes in the diagnostic criteria for what is now called substance use disorders. Briefly, four symptoms of substance abuse were combined with seven symptoms of substance dependence. In addition, the symptom of “desire” was included, and the “legal problems due to substance use” was removed. Thus eleven symptoms were listed for the diagnosis of substance use disorders. With the update of diagnostic criteria by DSM-5, updated versions of the original YFAS were published, namely YFAS 2.0 and mYFAS 2.0 [4]. Initially, it was believed that despite the extensive changes between the YFAS and the YFAS 2.0 and their versions, they would not present significant differences in determining the prevalence of FA, since both versions presented similar psychometric properties [4]. However, this fact does not seem to be supported by recent data from the systematic review by Praxedes et al. [2], in which the pooled prevalences of FA determined by mYFAS are lower than those determined by mYFAS 2.0 (7% versus 18%, respectively), highlighting the need for caution in interpreting the results of the study by Sultana et al. [1] again.

In the methods section, the authors report that they used the first version of mYFAS [5]. Considering that the diagnostic criteria for substance use disorder have undergone significant changes, it is noteworthy that Sultana et al. [1] used a version of the YFAS with outdated diagnostic criteria in a data collection carried out between February and March 2021. Thus, we reiterate the need for caution when considering the prevalence of FA found by them in university students in Bangladesh. Still, in this sense, Sultana et al. [1] report a prevalence of FA of 7.5% in their sample. A value much lower than the 19.1% of FA observed in a study conducted with a representative sample of university students in Brazil also during the COVID-19 pandemic [6] and also the pooled prevalence of FA (11%) in university students observed in the systematic review by Praxedes et al. [2].

## Conclusion

Thus, despite the importance and relevance of the study conducted by Sultana et al.[1] in Bangladesh. It is necessary to point out that the first version of the mYFAS must be considered when interpreting its results by readers and researchers in the area, given the important changes in the scale resulting from updating the diagnostic criteria for the disorders by substance use in the DSM-5.

## Data Availability

Not applicable.

## Abbreviations

DSM: Diagnostic and Statistical Manual of Mental Disorders
FA: food addiction
mYFAS: modified Yale Food Addiction Scale
YFAS: Yale Food Addiction Scale

## References

[1] Sultana MS, Islam MS, Sayeed A, Koly KN, Baker K, Hossain R, Ahmed S, Ferdous MZ, Mubarak M, Potenza MN, Sikder MT (2022). Food addiction, orthorexia nervosa and dietary diversity among Bangladeshi university students: a large online survey during the COVID-19 pandemic. J Eat Disord.

[2] Praxedes DRS, Silva-Júnior AE, Macena ML, Oliveira AD, Cardoso KS, Nunes LO, Monteiro MB, Melo ISV, Gearhardt AN, Bueno NB (2022). Prevalence of food addiction determined by the Yale Food Addiction Scale and associated factors: a systematic review with meta-analysis. Eur Eat Disord Rev.

[3] Gearhardt AN, Schulte EM (2021). Is food addictive? A review of the science. Annu Rev Nutr.

[4] Meule A, Gearhardt AN (2019). Ten years of the Yale Food Addiction Scale: a review of version 2.0. Curr Addict Rep.

[5] Flint AJ, Gearhardt AN, Corbin WR, Brownell KD, Field AE, Rimm EB (2014). Food-addiction scale measurement in 2 cohorts of middle-aged and older women. Am J Clin Nutr.

[6] da Silva Júnior AE, de Lima Macena M, de Oliveira ADS, Praxedes DRS, de Oliveira Maranhão Pureza IR, de Menezes Toledo Florêncio TM, Gearhardt AN, Bueno NB (2022). Prevalence of food addiction and its association with anxiety, depression, and adherence to social distancing measures in Brazilian universitystudents during the COVID-19 pandemic: a nationwide study. Eat Weight Disord.

